# Effects of *Blidingia* sp. Extract on Intestinal Inflammation and Microbiota Composition in LPS-Challenged Mice

**DOI:** 10.3389/fphys.2019.00763

**Published:** 2019-06-25

**Authors:** Wei Song, Yan Li, Xuelei Zhang, Zongling Wang

**Affiliations:** ^1^Key Laboratory of Science and Engineering for Marine Ecology and Environment, The First Institute of Oceanography, Ministry of Natural Resources of the People’s Republic of China, Qingdao, China; ^2^Laboratory of Marine Ecology and Environmental Science, Qingdao National Laboratory for Marine Science and Technology, Qingdao, China

**Keywords:** apoptosis, *Blidingia* sp., inflammation, microbiota, morphology

## Abstract

*Blidingia* sp. is a green alga that has spread rapidly in Subei Shoal, China. To explore the potential beneficial effects of *Blidingia* sp., we investigated the anti-inflammatory activity of its water–methanol extract of *Blidingia* sp. in a mouse model of lipopolysaccharide (LPS)-induced intestinal inflammation. The results revealed that the administration of *Blidingia* extract significantly alleviated the LPS-induced increase of the inflammatory cytokine content in the serum, as well as latter’s gene expression in the ileum. Moreover, the extract inhibited the phosphorylation of NF-κB and IκBα in LPS-challenged mice. Apart from these changes, the extract also averted intestinal morphology damage(s) and cell apoptosis in mice. Interestingly, the extract also had beneficial effects on the diversity and composition of caecal microbiota in LPS-challenged mice. In conclusion, the results suggested that *Blidingia* extract had beneficial effects on the recovery of intestinal function by reducing the inflammatory response, improving the maintenance of intestinal morphology, and decreasing cell apoptosis in LPS-induced intestinal inflammation. In addition, the beneficial effects of the extract on caecal microbiota composition may play a role in its anti-inflammatory activity. These results suggested that *Blidingia* extract could be potentially used in preventing intestinal inflammation.

## Introduction

Inflammation is an innate defense mechanism in response to tissue injury, stress, and infection. However, an excessive and acute inflammation could be potentially damaging. Moreover, a chronic and prolonged inflammation could promote the development of diabetes, obesity, cancer, and cardiovascular diseases (Wang Z. et al., 2017). The gastrointestinal tract is a primary site that faces exogenous materials and serves as a vital defense barrier against harmful substances and microbiota (Zhang H. et al., 2018). As a result, it is susceptible to inflammatory responses. Acute treatment of lipopolysaccharides (LPS), (produced by the Gram-negative bacterial cell wall) induces rapid accumulation of various cytokines that play an important role in the inflammatory process (Zhou et al., 2012, 2017b). Particularly, the use of an LPS-induced intestinal inflammation rodent model is common for evaluating the anti-inflammatory activity of natural products (Raetz and Whitfield, 2002).

Seaweeds, such as *Ulva prolifera*, have been used as a health food for ages due to their richness in polysaccharides, polyphenols, essential amino acids, and mineral elements (Song et al., 2018). Polysaccharides, flavonoids, polyphenols, and other components extracted from the seaweeds including *U. prolifera*, have exhibited anti-inflammatory effects since long (Okai and Higashi-Okai, 1997; Jung et al., 2013; Song et al., 2018). In addition, these extracts exhibit profound effects on the intestinal microbiota composition which is closely associated with the development of intestinal inflammatory response (Ren et al., 2018; Zhang Z. et al., 2018). *Blidingia* sp. is ubiquitous on several coastlines and often grows together with *Ulva* spp. (Woolcott et al., 2000). It is one of the dominant fouling green macroalgae in the *Pyropia* aquaculture facilities of Subei Shoal (Jiangsu, China) (Wang et al., 2015). Until now, little research has reported the effects of *Blidingia* extract on LPS-induced inflammation. To explore the beneficial effects of this extract, we scraped *Blidingia* sp. from the *Pyropia* aquaculture facilities for extraction. Furthermore, the components of the extract were analyzed through UHPLC-Q-Extractive-Orbitrap/MS and its effects on intestinal inflammation and microbiota composition were determined.

## Materials and Methods

### Sample Preparation

The green macroalgae *Blidingia* sp. was scraped from the *Pyropia* aquaculture facilities in the Subei Shoal. The collected *Blidingia* sp. was rinsed with sterile seawater and then dried in the sun. The air-dried powder of *Blidingia* sp. (1 kg) was extracted with 5 L volume of distilled water at 90°C for 3 h in an ultrasonic bath (200 W, 45 kHz). The supernatant was collected by filtering through the siliceous earth and then submitted to the adsorption chromatography column (120 cm L × 150 mm ID, Huamei Experiment Instrument Plant, Shanghai, China) filled with AB-8 macroporous adsorption resin. After eluted with distilled water at a flow rate of 60 mL/min, the extract was further eluted with 70% methanol. The small molecule mixture (BSE) was obtained by freeze-drying the eluent.

### UHPLC-Q-Extractive-Orbitrap/MS Analysis

First, 50 mg of BSE was dissolved in 1 mL of extract solvent (acetonitrile-methanol-water, 2:2:1, containing internal standard 1 μg/mL) and then centrifugated at 8,000°*g* at 4°C for 15 min. The resulting supernatants were transferred to LC-MS vials for UHPLC-QE-Orbitrap/MS analysis as previously described (Li et al., 2017). The analyses were performed using an 1290 UHPLC system (Agilent, Palo Alto, CA, United States) with a UPLC HSS T3 column (2.1 mm × 100 mm, 1.8 μm) coupled to Q Exactive (Orbitrap MS, Thermo, Somerset, NJ, United States). The mobile phase A was 0.1% formic acid in water for positive, and 5 mmol/L ammonium acetate in water for negative, and the mobile phase B was acetonitrile. The elution gradient was set as follows: 0 min, 1% B; 1 min, 1% B; 8 min, 99% B; 10 min, 99% B; 10.1 min, 1% B; and 12 min, 1% B. The flow rate was 0.5 mL/min and the injection volume was 2 μL.

### Animals and Treatment

Thirty nine-week-old male C57BL/6J mice were obtained from the SLAC Laboratory Animal Central (Changsha, China) and were acclimatized for 2 weeks under an environmental cycle of 12 h light/12 h dark. All animals were fed *ad libitum* and free to obtain water during the experiment. All mice were orally gavaged with either *Blidingia* sp. extract (BSE) (10 mg/kg body weight, *n* = 10) or the same volume of saline (*n* = 20) for 14 days. The dosage of *Blidingia* sp. extract used in the present study was based on our preliminary experiments. At 10:00 am on day 15, the mice gavaged with saline were challenged with intraperitoneally injection of either LPS (0.5 mg/kg, Escherichia coli serotype 055:B5; Sigma Chemical, Inc., St. Louis, MO, United States; *n* = 10) or saline (*n* = 10), while the mice gavaged with BSE were all challenged with LPS (0.5 mg/kg; *n* = 10). At 2 h after treatment with LPS or saline, samples of blood, ileum (1–2 cm proximal to the ileocecal valve) and caecal digesta were collected for further analysis. The experimental protocol was approved by the Protocol Management and Review Committee of the First Institute of Oceanography of China, and the mice were cared for and sacrificed according to the animal care guidelines of the First Institute of Oceanography of China.

### Determination of Inflammatory Cytokine Content in Serum

Tumor necrosis factor a (TNF-a), Interleukin 6 (IL-6), IL-8, and IL-10 content in serum were determined using ELISA quantitative kits (Cusabio Biotech, Wuhan, China) according to the manufacturer’s instructions.

### RT-qPCR Analysis

The ileum samples were used for total RNA extraction using TRIzol reagent (Invitrogen, Shanghai, China) and then cDNA was obtained using the PrimeScript RT reagent kit (Takara, Dalian, China) (Zhou et al., 2017a, 2018). RT-qPCR was performed in 10 μL assay volumes containing 3 μL of DEPC-treated H_2_O, 0.2 μL of ROX, 1 μL of cDNA template, 0.4 μL forward primer and 0.4 μL reverse primer, and 5 μL of SYBR Green mix (Takara). All samples were run in triplicate and the results were obtained by calculating the average values. The primer sequences are presented in Supplementary Table S1.

### Protein Qualification by the Wes Simple Western System

Protein expression were qualified using the Wes Simple Western System (Proteinsimple, San Jose, CA, United States). Proteins extracted from the ileum samples were mixed with Master Mix, dithiothreitol, fluorescent standards and Simple Western Sample Buffer (Proteinsimple) and then were loaded into Wes 25-well plates. Primary antibodies (β-actin, phopho NFκB, NFκB, phopho IκBα and IκBα, Abcam, Cambridge, MA, United States), secondary antibodies, luminol-peroxide mixture, stacking gel matrix, and separation gel matrix were added according to the manufacturer’s instructions. Results were collected using the “gel view” function of the Protein Simple software (Proteinsimple).

### Haematoxylin-Eosin (HE) Staining and Transmission Electron Microscopy

The ileum samples were opened longitudinally, fixed with 4% formaldehyde, and then embedded in paraffin. Thereafter, samples were sliced as sections with 8-μm thickness for HE staining (Yin et al., 2018). The histological scoring was performed based on previous description (Zhang H. et al., 2018). Meanwhile, the ileum samples were also fixed in 2.5% glutaraldehyde and post-fixed in osmium tetroxide. After washed with PBS, the ileum samples were dehydrated with graded alcohol and then embedded in Epon-Araldite resin. Finally, samples were sliced as ultrathin sections with 50-nm thickness, stained with uranyl acetate and lead citrate, and observed with a Zeiss 902 transmission electron microscope.

### Assessment of Apoptosis

The ileum samples were opened longitudinally, fixed with 10% formaldehyde, and then embedded in paraffin. Thereafter, samples were sliced as sections with 5-μm thickness for TUNEL staining using an *in situ* cell death detection kit (Roche, Shanghai, China). Nuclei were stained using DAPI mounting solution (Vector, Burlingame, CA, United States). Representative results were collected using a light microscope.

### Measurement of the Caecal Microbiota

Samples of all the content within the caecal digesta from treated mice were pooled and homogenized and then used for DNA extraction using the QIAamp DNA stool MiniKit (Qiagen, Shanghai, China). Bacterial 16S rRNA gene sequences (V3–V4 region) were amplified using specific primers with Premix Ex Taq^TM^ Hot Start Version (Takara, Dalian, China). A total volume of 50 mL consisting of 12.5 mL of Phusion High-Fidelity PCR Master Mix (New England BioLabs Inc., Beverly, MA, United States), 50 ng of template DNA, 1 mL of each primer, and PCR-grade water were mixed for the performance of PCR reaction. Then, MiSeq Illumina sequencing was performed on the sequencing reaction (Illumina Inc., San Diego, CA, United States) for paired-end reads. Following, the paired-end reads were assembled, merged and assigned to each sample based on the unique barcodes. Based on a 97% sequence similarity, high-quality tags were clustered into operational taxonomic units (OTUs), which were used for further analysis using database of Greengenes by RDP algorithm. The results of alpha and beta diversity and principal coordinate analysis (PCoA) were obtained using QIIME software. The results of linear discriminant analysis (LDA) effect size (LEfSe) were collected using the LEfSe tool.

### Statistical Analyses

All data were analyzed by one-way ANOVA using the general linear model procedures and a mixed procedure (PROCMIXED) of SAS software version 9.2 (SAS Institute Inc., Cary, NC, United States). Data are presented as least squares means ± SEM. Mean values were considered significantly different when *P* < 0.05.

## Results

### Characterization of Components of *Blidingia* sp. Extract

In total, over two hundred compounds were detected in the *Blidingia* sp. extract with the high-resolution UPLC-QE-Orbitrap/MS system (Supplementary Table S2). Twenty-eight species of amino acids, 15 species of nucleotides, 103 species of peptides, 49 species of organic acids, 10 species of alkaloids, 3 species of phenols, and other compounds were detected in positive ionization mode.

### *Blidingia* sp. Extract Alleviates Inflammatory Response in LPS-Challenged Mice

**FIGURE 1:**
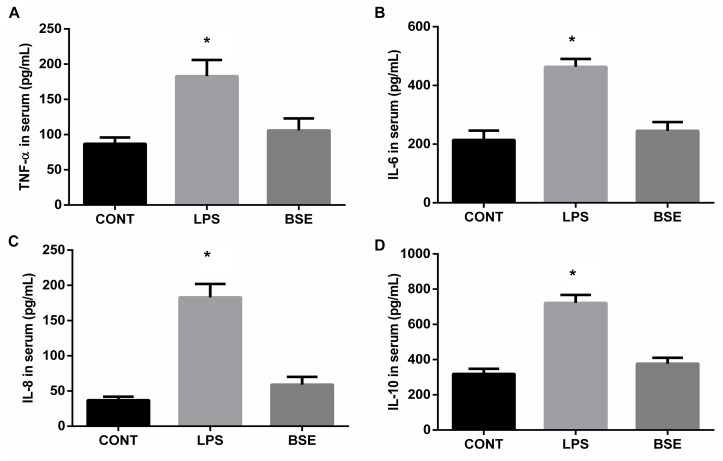
Effects of *Blidingia* sp. extract on serum inflammatory cytokines content in lipopolysaccharide-challenged mice. **(A)** TNF-α content; **(B)** IL-6 content; **(C)** IL-8 content; **(D)** IL-10 content. CONT, mice gavaged with sterile saline; LPS, mice injected with lipopolysaccharide; and BSE, mice gavaged with *Blidingia* sp. extract and injected with lipopolysaccharide. Values are expressed as mean±SEM, *n* = 8; ^∗^*p* < 0.05.

**FIGURE 2:**
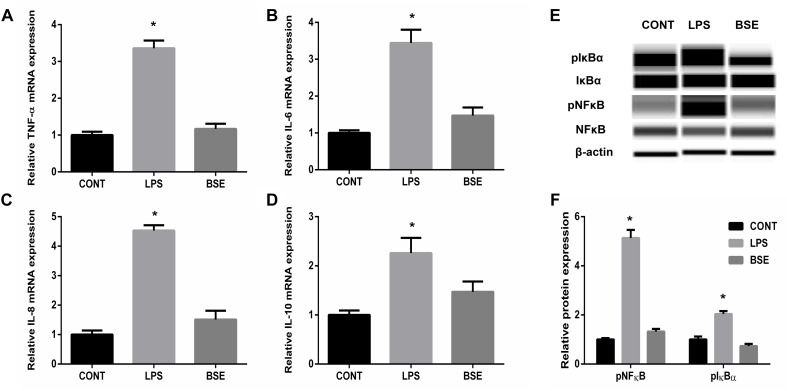
Effects of *Blidingia* sp. extract on expression of inflammatory cytokines and expression of proteins involved in NFκB pathway in ileum of lipopolysaccharide -challenged mice. **(A)** TNF-α mRNA expression; **(B)** IL-6 mRNA expression; **(C)** IL-8 mRNA expression; **(D)** IL-10 mRNA expression; **(E)** Western blotting results; **(F)** Relative abundance of pNFκB and pIκBα to total NFκB and IκBα protein expression, respectively. CONT, mice gavaged with sterile saline; LPS, mice injected with lipopolysaccharide; and BSE, mice gavaged with *Blidingia* sp. extract and injected with lipopolysaccharide. Values are expressed as mean±SEM, *n* = 8 for qRT-PCR and *n* = 3 for Western blotting; ^∗^*p* < 0.05.

LPS challenge induced significant increases of TNF-a, IL-6, IL-8, and IL-10 contents in serum, while *Blidingia* sp. extract significantly decreases their contents (Figure 1). Moreover, mRNA expression of TNF-a, IL-6, IL-8, and IL-10 in ileum were also increased. However, administration of *Blidingia* sp. extract alleviated these LPS-induced changes (Figure 2A–D). Additionally, expression of phosphorylated NFκB and IκBα in LPS-challenged mice were significantly higher when compared with control mice, while no significant difference was observed in their expression between control mice and mice administrated with *Blidingia* sp. extract (Figure 2E,F) (Supplementary Figures S1–S4).

### *Blidingia* sp. Extract Alleviates LPS-Induced Histopathological Changes and Apoptosis

**FIGURE 3:**
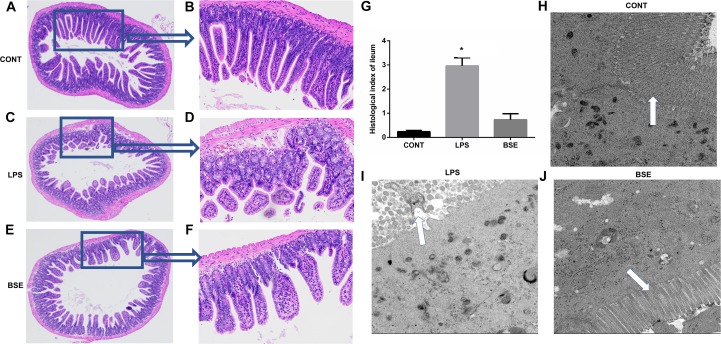
Effects of *Blidingia* sp. extract on ileum morphology in lipopolysaccharide – challenged mice. **(A–F)** HE staining of ileum morphology (**A,C,E**, 100 ×; **B,D,F**, 40 ×); **(G)** Histological index of ileum; **(H–J)** ultrastructural observation of microvilli in ileum (transmission electron microscopy, 5,000 ×). CONT, mice gavaged with sterile saline; LPS, mice injected with lipopolysaccharide; and BSE, mice gavaged with *Blidingia* sp. extract and injected with lipopolysaccharide. Values are expressed as mean±SEM, *n* = 3; ^∗^*p* < 0.05.

**FIGURE 4:**
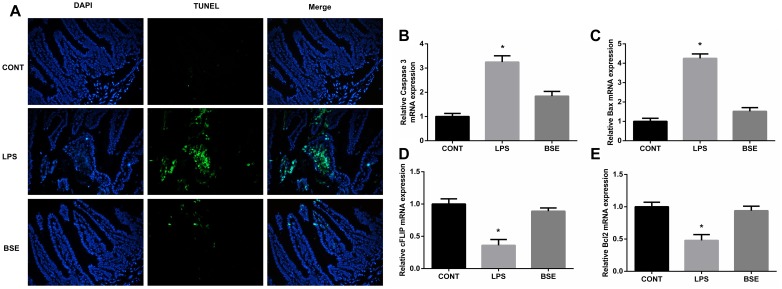
Effects of *Blidingia* sp. extract on apoptosis in ileum of lipopolysaccharide – challenged mice. **(A)** Representative TUNEL (green) staining (200 ×); Nuclei were stained with DAPI (blue). Relative mRNA expression of Caspase 3 **(B)**, Bax **(C)**, cFLIP **(D)**, and Bcl2 **(E)**. CONT, mice gavaged with sterile saline; LPS, mice injected with lipopolysaccharide; and BSE, mice gavaged with *Blidingia* sp. extract and injected with lipopolysaccharide. Values are expressed as mean±SEM, *n* = 8; ^∗^*p* < 0.05.

The HE staining results showed pathological changes, such as shedding and obvious edema, following LPS challenge in the ileum tissue, while no such changes were observed in control mice and mice administrated with *Blidingia* sp. extract (Figure 3A–F). Mice challenged with LPS had a significant higher histological index of ileum when compared with control mice or mice administrated with *Blidingia* sp. extract (Figure 3G). Moreover, the TEM results showed that irregularly arranged microvilli were only observed in ileum of LPS-challenged mice (Figure 3H–J). TUNEL staining revealed that the level of apoptosis was higher in LPS-challenged mice when compared with control mice, while no difference was observed between control mice and mice administrated with *Blidingia* sp. extract (Figure 4A). Moreover, LPS challenge induced significant increases of mRNA expression of Bax and Caspase 3, while significant decreases of mRNA expression of cFLIP and Bcl2 (Figure 4B–E). However, administration of *Blidingia* sp. extract alleviated these LPS-induced changes.

### Effects of *Blidingia* sp. Extract on Microbial Diversity

**FIGURE 5:**
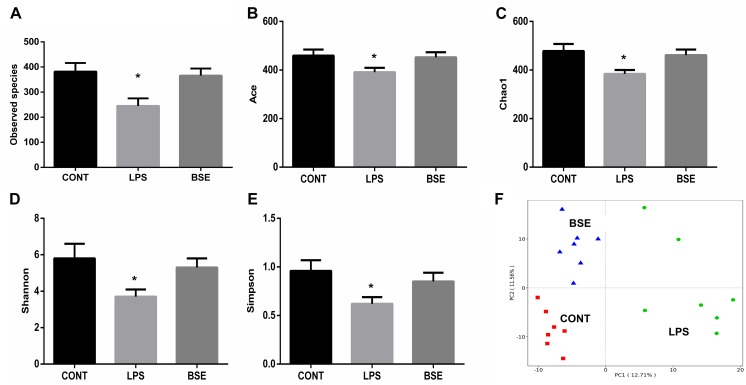
Effects of *Blidingia* sp. extract on caecal microbiota diversity in lipopolysaccharide – challenged mice. **(A)** Observed species; **(B)** Ace; **(C)** Chao1; **(D)** Shannon; **(E)** Simpson; **(F)** PCoA plot of the microbiota based on an unweighted UniFrac metric. CONT, mice gavaged with sterile saline; LPS, mice injected with lipopolysaccharide; and BSE, mice gavaged with *Blidingia* sp. extract and injected with lipopolysaccharide. Values are expressed as mean±SEM, *n* = 7; ^∗^*p* < 0.05.

Based on the V3 + V4 region of the 16D rDNA sequence, an average of 80,012 (71,129-86,714) effective tags were used for the analysis of the study, OTUs were generated from sequences with at least 97% similarity. Alpha diversity including Observed species, Ace, Chao1, Shannon and Simpson index, as well as weight PCoA analysis were measured to detect the diversity and structure of caecal microbial communities in mice after treatment of LPS and BSE. The alpha diversity of microbial communities, as indicated by the index of Observed species, Ace, Chao1, Shannon and Simpson, was decreased significantly by the treatment of LPS, while BSE supplementation significantly increased the microbial diversity (Figure 5A–E). In addition, the PCoA plot based on the weighted UniFrac metric showed that caecal microbiota was significantly regulated by LPS and BSE treatment (Figure 5F).

### Effects of *Blidingia* sp. Extract on Microbial Compositions

**FIGURE 6:**
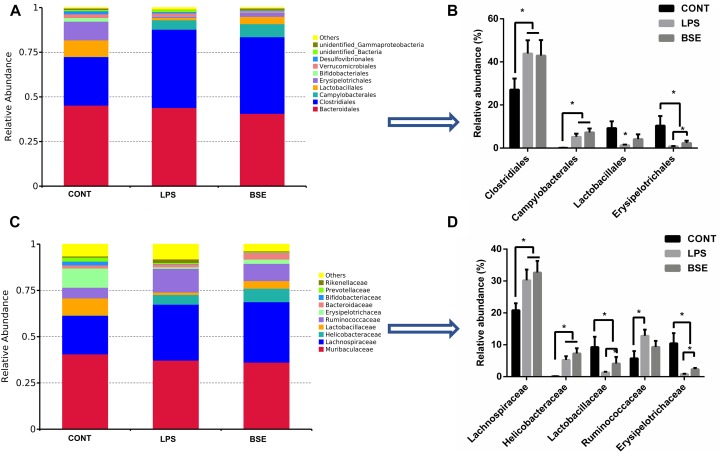
Effects of *Blidingia* sp. extract on relative abundance of predominant bacteria at the order and family level in cecum of lipopolysaccharide-challenged mice. Relative abundance of predominant bacteria at the class **(A,B)**, and family **(C,D)** level. CONT, mice gavaged with sterile saline; LPS, mice injected with lipopolysaccharide; and BSE, mice gavaged with *Blidingia* sp. extract and injected with lipopolysaccharide. Values are expressed as mean±SEM, *n* = 7; ^∗^*p* < 0.05.

**FIGURE 7:**
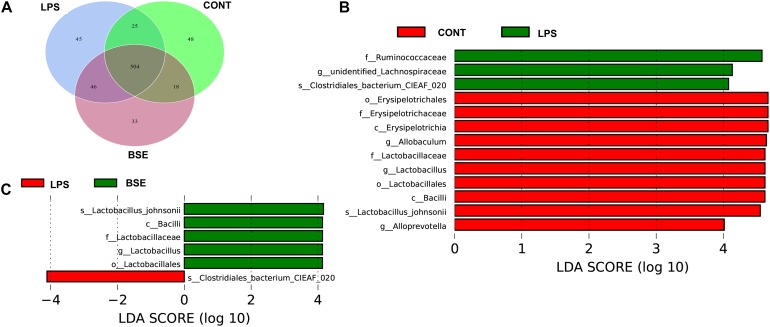
Effects of *Blidingia* sp. extract on microbial community composition in cecum of lipopolysaccharide – challenged mice. **(A)** Venn diagram of OTUs; **(B,C)** Taxa value meeting a significant LDA threshold value of >4.0 are shown, which are displayed with a positive LDA score. CONT, mice gavaged with sterile saline; LPS, mice injected with lipopolysaccharide; and BSE, mice gavaged with *Blidingia* sp. extract and injected with lipopolysaccharide.

The order level analysis demonstrated that the percentage of *Clostridiales* and *Campylobacterales* were significantly increased in the cecum of mice after the treatment of LPS, while BSE treatment had no effects on these increases (Figure 6A,B); LPS treatment also caused significant decreases of percentage of *Lactobacillales* and *Erysipelotrichales*, while BSE treatment alleviated these decreases (Figure 6A,B). In the class level, the percentage of *Lachnospiraceae* and *Helicobacteraceae* were significantly increased after the treatment of LPS, while BSE treatment had no effects on these increases (Figure 6C,D); LPS treatment also caused significant decreases of percentage of *Lactobacillaceae* and *Erysipelotrichaceae*, as well as an increase of *Ruminococcaceae*, while BSE treatment alleviated these changes in some extent (Figure 6C,D). The Venn diagram, revealing the overlapping OTUs data, displaying that 504 OTUs were universal to all samples, and there are 48 unique OTUs in control mice, 45 unique OTUs in LPS-treated mice and 33 unique OTUs in BSE-treated mice (Figure 7A).

To identify the bacterial taxa associated with the beneficial effects by BSE treatment, we used a LEfSe analysis to compare caecal microbiota (Figure 7B,C). A significant difference in the relative abundance was determined when LDA score >4. We observed higher relative abundance of *Bacilli*, as well as its lower taxa *Lactobacillales* and *Lactobacillaceae* in both Control and BSE group when compared with the LPS group. In addition, a higher relative abundance of *Erysipelotrichaceae*, as well as its lower taxa *Erysipelotrichales* and *Erysipelotrichaceae* were also observed in Control group. The LPS group showed higher abundance of *Ruminococcaceae* in the family level, *Clostridia* in the class level and *Clostridiales* in the order level when compared with the Control group.

## Discussion

The green alga, *Blidingia* sp., is widely distributed in Subei Shoal, China. In the present study, *Blidingia* sp. was collected and the effects of its extract on the intestinal inflammatory response and caecal microbiota composition were evaluated. It was observed that *Blidingia* sp. extract decreased the inflammatory cytokine content and inhibited the activation of the NF-κB signaling pathway in a mouse model of LPS-induced intestinal inflammation. The extract is majorly constituted of amino acids, polypeptides, organic acids, phenols, flavonoids, nucleotides, and alkaloids. These components are speculated to play critical effects on its anti-inflammatory activity. LPS-induced inflammation is associated with intestinal morphological damages and elevated apoptosis levels that act as key indicators in characterizing the dysfunctional condition of intestine. These changes were, however, not observed after the mice were administrated with the *Blidingia* extract. This observation suggested that the extract could have supported intestinal function by protecting the intestine from morphological damage and decreasing cell apoptosis besides alleviating the inflammatory response.

High diversity of intestinal microbiota is suggested as more stable and healthier (Konstantinov et al., 2004), whereas, human patients and animal models with intestinal inflammation displayed reduced bacterial species diversity (Wang K. et al., 2017; Zhang H. et al., 2018). In the present study, the results proposed that LPS treatment not only decreases the community richness (Chao1 and ACE indices) but also community diversity (Shannon and Simpson indices) of the caecal microbiota. In addition, PCoA analysis revealed a shift in the bacterial community composition. These results were corroborated by a previous study on LPS-challenged piglet model (Wang et al., 2019). Importantly, LPS treatment caused a significant increase in the bacterial family *Ruminococcaceae*, that harbors potential pathogen bacteria found in other intestinal inflammatory models (Wang et al., 2019). On the other hand, LPS treatment marked a decrease of the order Lactobacillales that is comprised of the lactic acid bacteria with well-known probiotic properties (Ritchie et al., 2015). The results indicated that LPS accounted for intestinal inflammation in association with the disruption of caecal microbiota. In addition, LPS treatment resulted in a significant increase of Clostridiales (at the order level) and *Lachnospiraceae* (at the family level) both of which are bacterial subclasses of the phylum Firmicutes. *Ruminococcaceae* and *Lachnospiraceae* are the most abundant Firmicute families observed in the intestine environments (Tap et al., 2009). *Lachnospiraceae* has a beneficial effect on the health of intestinal epithelial tissue as it is documented to be associated with butyrate production (Barcenilla et al., 2000). However, reduction of *Ruminococcaceae* and *Lachnospiraceae* has been reported in patients with Crohn’s disease or inflammatory bowel disease, which are in agreement with our results (Frank et al., 2007; Fujimoto et al., 2013). The result changes were presumed as an immediate positive response of the caecal microbiota to LPS induced inflammation. However, the exact explanation of these changes needs to be further elucidated.

Recently, many studies have revealed that polysaccharides, flavonoids, and polyphenols extracted from the green seaweeds such as *Porphyra haitanensis* and *U. prolifera* had potential effects on the intestinal microbiota (Zhang Z. et al., 2018; Lin et al., 2019; Yan et al., 2019). In the present study, the effects of *Blidingia* sp. extract on the caecal microbiota of LPS-treated mice were assessed. The results displayed beneficial effects as the extract increased the bacterial diversity. Importantly, *Blidingia* extract increased the population of Bacilli, as well as its lower taxa; Lactobacillales, *Lactobacillaceae*, and *Lactobacillus* in LPS-treated mice. These effects were in agreement with a previous report on *Enteromorpha clathrata* extracts (Shang et al., 2018). However, *Blidingia* extract displayed no effects on Clostridiales and Campylobacterales (at the order level), as well as *Lachnospiraceae* and *Helicobacteraceae* (at the family level). Nevertheless, the results suggested a prebiotic effect of the extract on caecal microbiota of the mouse model with LPS-induced inflammation.

## Conclusion

In conclusion, the results of this study indicated that *Blidingia* extract has beneficial effects on the recovery of intestinal function by alleviating the inflammatory response, improving the maintenance of intestinal morphology, and decreasing cell apoptosis in a mouse model of LPS-induced intestinal inflammation. In addition, the extract also exerted positive effects on caecal microbiota diversity and composition, which may play a role in its anti-inflammatory activity. The results suggested the potential use of *Blidingia* extract in preventing intestinal inflammation.

## Data Availability

The raw data supporting the conclusions of this manuscript will be made available by the authors, without undue reservation, to any qualified researcher.

## Ethics Statement

This study was carried out in accordance with the recommendations of the Protocol Management and Review Committee of the First Institute of Oceanography of China. The protocol was approved by the Animal Care Guidelines of the First Institute of Oceanography of China.

## Author Contributions

WS and ZW conceived and designed the research. WS, YL, and XZ performed all the protocol. WS, YL, ZW, and XZ wrote and revised the manuscript.

## Conflict of Interest Statement

The authors declare that the research was conducted in the absence of any commercial or financial relationships that could be construed as a potential conflict of interest.

## Supplementary Material

FIGURE S1 Complete protein band of IκBα and pIκBα.

FIGURE S2 Complete protein band of pNFκB.

FIGURE S3 Complete protein band of β-actin.

FIGURE S4 Complete protein band of NFκB.

TABLE S1 Primer sequences for RT-PCR.

TABLE S2 Characterization of components of *Blidingia* sp. extract by UHPLC-Q-Extractive-Orbitrap/MS Analysis.
