# Supplementary figures and images for: Effects of Blidingia sp. Extract on Intestinal Inflammation and Microbiota Composition in LPS-Challenged Mice
Source: Front Physiol. 2019 Jun 25;10:763. doi: 10.3389/fphys.2019.00763 (PMC6603216; doi:10.3389/fphys.2019.00763)

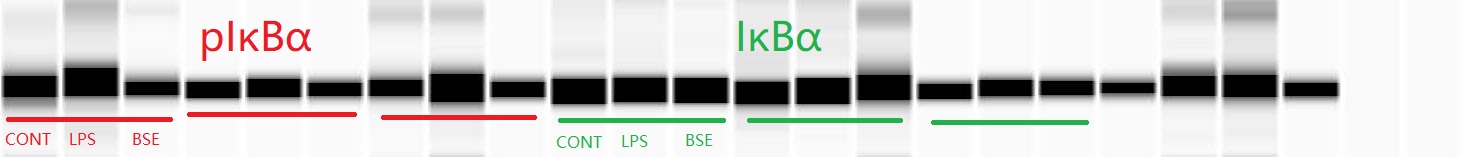

Supplement: FIGURE S1 — Complete protein band of IκBα and pIκBα. [file Image_1.JPEG]

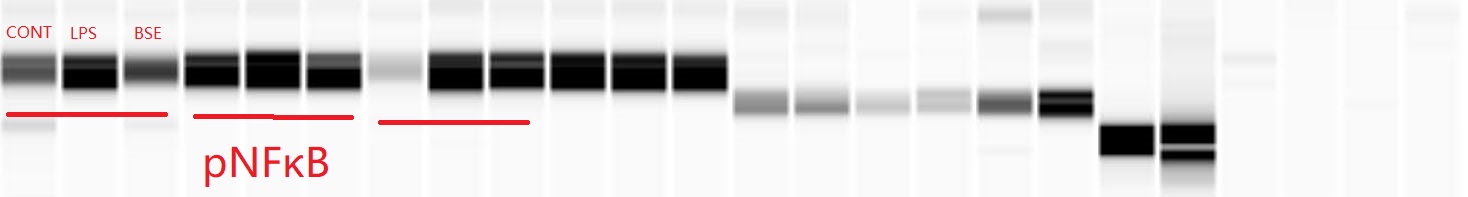

Supplement: FIGURE S2 — Complete protein band of pNFκB. [file Image_2.JPEG]

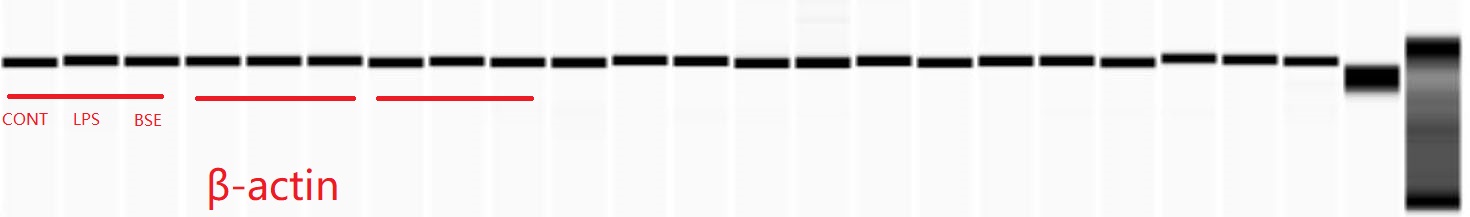

Supplement: FIGURE S3 — Complete protein band of β-actin. [file Image_3.JPEG]

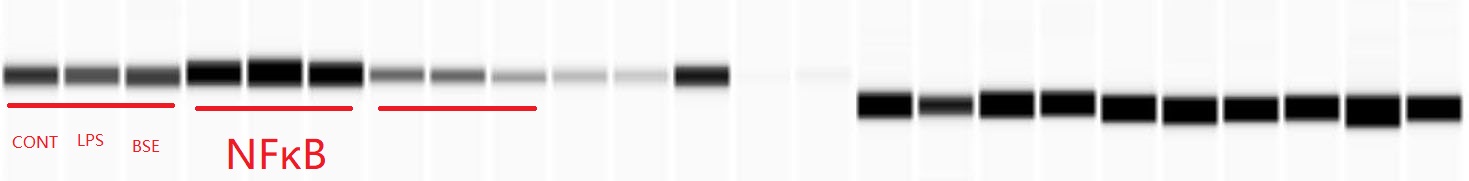

Supplement: FIGURE S4 — Complete protein band of NFκB. [file Image_4.JPEG]
