# Supplementary material for: Effects of Blidingia sp. Extract on Intestinal Inflammation and Microbiota Composition in LPS-Challenged Mice
Source: Front Physiol. 2019 Jun 25;10:763. doi: 10.3389/fphys.2019.00763 (PMC6603216; doi:10.3389/fphys.2019.00763)
Supplement: TABLE S1 — Primer sequences for RT-PCR. [file Table_1.DOCX]

**Supplementary Table 1** Primer sequences for RT-PCR

| Gene | 5’-3’ Primer sequence |
| --- | --- |
| TNF-α | F: ATGAGAAGTTCCCAAATGGC |
|  | R: CTCCACTTGGTGGTTTGCTA |
| IL-6 | F: CCTCTCTGCAAGAGACTTCCAT |
|  | R: AGTCTCCTCTCCGGACTTGT |
| IL-8 | F: CTAGGCATCTTCGTCCGTCC |
|  | R: CAGAAGCTTCATTGCCGGTG |
| IL-10 | F: GGACCAGCTGGACAACATACTGCTA |
|  | R: CCGATAAGGCTTGGCAACCCAAGT |
| Bax | F: CTGGGCCTCCTCTCCTACT |
|  | R: CCTTTCCCCTTCCCCCATTC |
| Caspase 3 | F: GTGCGGTGTCCCTAAACCT |
|  | R: GAGTCCACTGACTTGCTCCC |
| cFLIP | F: CCGGACAGAGTGTCTCTATTG |
|  | R: TTCTCGTCCAGTCTCCATCC |
| Bcl2 | F: GAACTGGGGGAGGATTGTGG |
|  | R: GCATGCTGGGGCCATATAGT |
| β-actin | F: TGTCCACCTTCCAGCAGATGT |
|  | R: AGCTCAGTAACAGTCCGCCTAGA |

**Supplementary Table 2** Characterization of components of *Blidingia sp* extract by UHPLC-Q-Extractive-Orbitrap/MS Analysis

| **Proposed compounds** | **[M]+ (m/z)** | | **RT (min)** | **MS score** |
| --- | --- | --- | --- | --- |
| L-Valine | 118.0861 | | 42.0477 | 0.99680 |
| Isoleucine | 132.1018 | | 92.8915 | 0.99650 |
| Tyrosine | 182.0811 | | 131.6280 | 0.99584 |
| Asparagine | 133.0606 | | 35.0475 | 0.99513 |
| Citrulline | 176.1109 | | 36.5285 | 0.99463 |
| Arginine | 175.1187 | | 32.8018 | 0.99395 |
| Methionine | 150.0582 | | 45.4808 | 0.99220 |
| alpha-Methyl-DL-serine | 120.0654 | | 32.7871 | 0.99173 |
| Pyroglutamic acid | 130.0498 | | 56.6364 | 0.99004 |
| Acetyl-L-tyrosine | 224.0915 | | 150.6850 | 0.98970 |
| Leu | 132.1018 | | 33.8168 | 0.98878 |
| Prolyl-Tyrosine | 279.1334 | | 157.1340 | 0.98873 |
| L-prolyl-L-glycine | 173.0920 | | 157.1130 | 0.98735 |
| Valyl-Arginine | 274.1869 | | 144.9655 | 0.98623 |
| Glutamic acid | 148.0602 | | 31.4941 | 0.98622 |
| Glutaminyl-Proline | 244.1291 | | 167.9550 | 0.98468 |
| Phenylalanyl-Isoleucine | 279.1699 | | 216.4480 | 0.98374 |
| Prolyl-Arginine | 272.1712 | | 40.3079 | 0.98276 |
| Glycyl-Gamma-glutamate | 204.0976 | | 71.9056 | 0.97908 |
| L-Glutamine | 147.0763 | | 43.2628 | 0.97897 |
| Leucyl-Histidine | 269.1628 | | 185.3005 | 0.97259 |
| Threoninyl-Asparagine | 234.1121 | | 43.7880 | 0.97246 |
| L-beta-aspartyl-L-phenylalanine | 281.1129 | | 178.5090 | 0.95786 |
| Alanyl-Arginine | 246.1555 | | 109.7410 | 0.95649 |
| Proline | 116.0705 | | 35.5412 | 0.99999 |
| Tryptophan | 205.0970 | | 172.295 | 0.99904 |
| Homoserine | 120.0654 | | 59.0261 | 0.99779 |
| Asn Leu His | 383.2029 | | 249.1870 | 0.99915 |
| Gly Pro Asp Pro | 385.1742 | | 354.7090 | 0.99876 |
| Phe Gly | 223.1075 | | 155.2040 | 0.99873 |
| Phe Thr | 267.1334 | | 148.6900 | 0.99869 |
| Leu Thr | 233.1492 | | 82.5975 | 0.99851 |
| Asp Phe Val | 380.1847 | | 461.1650 | 0.99844 |
| Ser His Asp | 358.1389 | | 240.6520 | 0.99729 |
| Met Glu Gly | 336.1224 | | 203.6290 | 0.99705 |
| Val Val Gly | 274.1758 | | 218.0620 | 0.99696 |
| Glycylproline | 173.0919 | | 174.4305 | 0.99628 |
| Pro Ala Gln | 315.1725 | | 197.7175 | 0.99611 |
| Val Arg Gly | 331.2080 | | 40.3079 | 0.99609 |
| Leu Gln | 260.1600 | | 96.4804 | 0.99600 |
| Asp Arg Ala Lys | 489.2793 | | 286.3460 | 0.99593 |
| Leu Leu Gly Lys | 430.3012 | | 169.4750 | 0.99587 |
| Leu Lys Ser | 347.2296 | | 277.9150 | 0.99586 |
| Gly Ala Pro | 244.1289 | | 146.2370 | 0.99561 |
| Asp Tyr | 297.1095 | | 202.0980 | 0.99518 |
| Cys Glu | 251.0698 | | 228.2610 | 0.99511 |
| Gly Ile Val | 288.1912 | | 233.3350 | 0.99500 |
| Ala Glu Leu | 332.1807 | | 220.4840 | 0.99474 |
| Gly Glu | 205.0817 | | 169.7470 | 0.99447 |
| Leu Ile | 245.1857 | | 201.8385 | 0.99386 |
| Tyr Gly | 239.1023 | | 161.7150 | 0.99367 |
| Pro Asn Gly | 287.1233 | | 155.3545 | 0.99346 |
| Lys Val Leu | 359.2644 | | 154.6380 | 0.99338 |
| Gly-Leu | 189.1233 | | 197.0700 | 0.99330 |
| Gly Phe | 223.1075 | | 175.9170 | 0.99285 |
| Ile Arg | 288.2026 | | 139.7655 | 0.99271 |
| Gly Gly Gly Gly | 247.1039 | | 167.3525 | 0.99269 |
| Lys Trp Arg Arg | 645.3953 | | 331.4670 | 0.99254 |
| Tyr Asp Gly | 354.1289 | | 160.1600 | 0.99191 |
| Leu Glu Leu | 374.2277 | | 216.7920 | 0.99190 |
| Lys His | 284.1720 | | 469.8735 | 0.99131 |
| Leu Ser Gln | 347.1917 | | 83.6473 | 0.99072 |
| Ile Asp Gln | 375.1865 | | 102.2090 | 0.99064 |
| Leu Lys | 260.1964 | | 44.0873 | 0.99018 |
| Ala Leu Ile | 316.2224 | | 241.2150 | 0.98980 |
| Val Lys | 246.1810 | | 154.6380 | 0.98939 |
| Leu Asp Asn | 361.1710 | | 57.2960 | 0.98925 |
| Ala Ala Pro | 258.1445 | | 36.0348 | 0.98869 |
| Asn Glu Ile | 375.1866 | | 143.1905 | 0.98858 |
| Glu-Val | 247.1327 | | 206.0445 | 0.98849 |
| Ile Lys Gly | 317.2176 | | 40.5547 | 0.98841 |
| Gly Gly Ala Ile | 317.1812 | | 56.6559 | 0.98805 |
| Val Glu | 247.1285 | | 45.4033 | 0.98637 |
| Gln Gly | 204.0976 | | 34.0603 | 0.98493 |
| Cyclo(L-Phe-L-Pro) | 245.1281 | | 231.6860 | 0.98467 |
| Met Met Gly Val | 437.1868 | | 148.6900 | 0.98423 |
| Lys Leu Val | 359.2645 | | 175.0570 | 0.98387 |
| Ala Tyr | 253.1178 | | 60.1290 | 0.98374 |
| Leu Ala Lys | 331.2329 | | 89.0141 | 0.98317 |
| Ala Pro Ile | 300.1912 | | 268.0290 | 0.98316 |
| Ile Pro Asn | 343.1968 | | 179.8780 | 0.98300 |
| Ile Val Val Ala | 401.2750 | | 227.3930 | 0.98216 |
| Met Gly Trp Glu | 522.2015 | | 33.8855 | 0.98141 |
| Lys Ile | 260.1965 | | 124.5440 | 0.98053 |
| Ile Tyr Val Trp | 580.3085 | | 257.7905 | 0.98013 |
| Glu Ile Val Asp | 475.2386 | | 246.9530 | 0.97995 |
| Leu Glu Ser | 348.1759 | | 126.8700 | 0.97995 |
| Pro Phe | 263.1396 | | 156.2410 | 0.97926 |
| Asn Ser Thr | 321.1342 | | 33.9680 | 0.97858 |
| Ala Ala Ala | 232.1287 | | 85.2761 | 0.97702 |
| Ser Pro Val | 302.1704 | | 192.0035 | 0.97698 |
| Val Pro Thr | 316.1860 | | 222.2520 | 0.97687 |
| Ile Pro | 229.1545 | | 206.6490 | 0.97640 |
| Arg Val Ala | 345.2236 | | 71.9419 | 0.97535 |
| Ile Val Pro | 328.2221 | | 322.7160 | 0.97503 |
| Leu Val Ala | 302.2069 | | 173.3530 | 0.97476 |
| Leu Gly Ala Gly | 317.1813 | | 136.7980 | 0.97420 |
| Val Tyr | 281.1492 | | 161.5870 | 0.97215 |
| Ala Phe Ala | 308.1650 | | 174.8555 | 0.97175 |
| Ala Gly Pro | 244.1234 | | 216.5170 | 0.97168 |
| Ile Ser Lys Phe | 494.2960 | | 197.2390 | 0.97056 |
| Glu Ile | 261.1442 | | 176.1615 | 0.97026 |
| Pro Phe Thr | 364.1859 | | 186.7480 | 0.96955 |
| Phe Lys | 294.1806 | | 98.0472 | 0.96914 |
| Lys Lys Gly Thr | 433.2799 | | 349.5140 | 0.96883 |
| Gly Val Pro Gly | 329.1812 | | 167.8885 | 0.96821 |
| Pro Ala Phe Ala | 405.2123 | | 214.1400 | 0.96800 |
| Val Ser | 205.1181 | | 138.9110 | 0.96769 |
| Val Asp Ile | 346.1964 | | 195.9900 | 0.96751 |
| Gln Glu Thr | 377.1657 | | 127.0790 | 0.96745 |
| Ile Ala Phe | 350.2065 | | 230.5325 | 0.96617 |
| Pro Pro Asn | 327.1656 | | 162.2170 | 0.96387 |
| Pro Val | 215.1388 | | 125.2660 | 0.96311 |
| Pro Glu | 245.1129 | | 39.6884 | 0.96268 |
| Leu Pro Pro Ile | 439.2907 | | 200.2090 | 0.96224 |
| Ile Lys | 260.1965 | | 152.6610 | 0.96136 |
| Cys His Lys | 387.1793 | | 348.3990 | 0.96082 |
| Ile Asn Ser | 333.1757 | | 159.8960 | 0.96011 |
| Val Thr Ser | 306.1653 | | 131.9070 | 0.96004 |
| Asn Val Ser Thr | 420.2077 | | 35.8196 | 0.95937 |
| Leu Lys Ala | 331.2331 | | 245.7030 | 0.95923 |
| Gln Lys | 275.1730 | | 155.3215 | 0.95918 |
| Val Leu Gly | 288.1912 | | 173.9110 | 0.95916 |
| Pro Ala Leu Ala | 371.2281 | | 214.3900 | 0.95860 |
| Gly Pro Ile | 286.1757 | | 203.8700 | 0.95663 |
| Pro His | 253.1292 | | 164.6180 | 0.95469 |
| Lys Ser Asp | 349.1708 | | 103.5605 | 0.95451 |
| Gly Asn Leu | 303.1659 | | 134.3810 | 0.95123 |
| Ile Pro Thr | 330.2017 | | 171.0955 | 0.94971 |
| Ile Asn Val | 345.2126 | | 187.5305 | 0.94704 |
| Uracil | 113.0344 | | 44.0348 | 0.99945 |
| Adenine | 136.0617 | | 43.4887 | 0.99912 |
| 6-Dimethylaminopurine | 164.0930 | | 144.7540 | 0.99818 |
| Ectoine | 143.0814 | | 35.9323 | 0.99270 |
| Hypoxanthine | 137.0457 | | 56.9486 | 0.98700 |
| cytosine | 112.0504 | | 614.7340 | 0.98476 |
| guanine | 152.0566 | | 134.1980 | 0.95712 |
| Deoxycytosine | 583.7525 | | 98.0712 | 0.99844 |
| Pyrimidine | 81.0447 | | 640.2600 | 0.95045 |
| Adenosine | 268.1035 | | 131.1120 | 0.99725 |
| Guanosine | 284.0955 | | 154.7110 | 0.99196 |
| deoxyguanosine 5'-monophosphate | 348.0695 | | 127.4490 | 0.98736 |
| Succinoadenosine | 384.1141 | | 157.0635 | 0.96771 |
| N-Formyl-4-amino-5-aminomethyl-2-methylpyrimidine | 167.0926 | | 571.6265 | 0.94909 |
| Guanosine 5'-monophosphate | 364.0643 | | 117.5040 | 0.99068 |
| 1-Aminocyclohexanecarboxylic acid | 144.1018 | | 56.3237 | 0.99941 |
| Ritalinic acid | 220.1370 | | 157.0635 | 0.99919 |
| LARIXINIC ACID | 127.0389 | | 170.8210 | 0.99906 |
| 3-Furoic acid | 113.0232 | | 38.2186 | 0.99847 |
| Stearidonic Acid | 277.2156 | | 276.4345 | 0.99749 |
| KOJIC ACID | 143.0338 | | 41.5741 | 0.99698 |
| 9,12-Octadecadiynoic Acid | 277.2157 | | 354.9580 | 0.99814 |
| Aminodiphenylacetic acid | 228.0997 | | 184.7290 | 0.99813 |
| 1-Aminocyclohexanecarboxylic acid | 144.1018 | | 56.3237 | 0.99941 |
| 9-cis-Retinoic acid | 301.2155 | | 433.3060 | 0.99598 |
| 4-guanidinobutanoic acid | 146.0923 | | 43.4887 | 0.99536 |
| 10-fluoro-capric acid | 191.1428 | | 262.6140 | 0.99504 |
| t-4-AMINOCROTONIC ACID | 102.0549 | | 160.1600 | 0.99503 |
| 12-Oxo-2,3-dinor-10,15-phytodienoic acid | 265.1793 | | 257.6530 | 0.99479 |
| (R)-(+)-2-Pyrrolidone-5-carboxylic acid | 130.0498 | | 31.2496 | 0.99178 |
| Methyl 2-aminobenzoate | 152.0705 | | 233.5735 | 0.99054 |
| 8-oxo-9,11-octadecadiynoic acid | 291.1948 | | 370.3100 | 0.99034 |
| o-Anisic acid | 153.0540 | | 133.9410 | 0.98962 |
| 4-t-Butylbenzoic acid | 179.1065 | | 235.2380 | 0.98856 |
| 12,13-Epoxy-9,15-octadecadienoic acid | 295.2261 | | 336.0850 | 0.98780 |
| L-1,2,3,4-Tetrahydro-beta-carboline-3-carboxylic acid | 217.0925 | | 164.9440 | 0.98589 |
| 20-hydroxy-5Z,8Z,11Z,14Z-eicosatetraenoic acid | 321.2417 | | 300.4350 | 0.98462 |
| 12,13S-epoxy-9Z,11,15Z-octadecatrienoic acid | 293.2104 | | 306.9430 | 0.98437 |
| 4-Deoxytetronic acid | 87.0440 | | 129.2300 | 0.98380 |
| Caffeic acid | 181.0494 | | 197.2930 | 0.98131 |
| 9S,10-epoxy-10,12Z-octadecadienoic acid | 295.2262 | | 384.3555 | 0.98104 |
| Salvianolic acid A | 495.1274 | | 195.9510 | 0.97953 |
| Norecasantalic acid | 181.1220 | | 283.9690 | 0.97919 |
| Acoric acid | 269.1742 | | 315.9245 | 0.97813 |
| 8-hydroxy-13Z-octadecene-9,11-diynoic acid | 291.1948 | | 292.2370 | 0.97765 |
| 8-hydroxy-13,17-octadecadiene-9,11-diynoic acid | 289.1791 | | 272.3915 | 0.97753 |
| 6-pentadecyl Salicylic Acid | 349.2727 | | 402.0790 | 0.97740 |
| L-trans-4-Methyl-2-pyrrolidinecarboxylic acid | 130.0862 | | 44.0873 | 0.97291 |
| (alpha)-4-Methylene-2-pyrrolidinecarboxylic acid | 128.0706 | | 173.3640 | 0.97274 |
| L-Hexahydro-3-imino-1,2,4-oxadiazepine-3-carboxylic acid | 160.0716 | | 205.5930 | 0.97262 |
| Avenic acid A | 323.1441 | | 73.4930 | 0.97171 |
| 2-Aminoisobutyric acid | 104.0705 | | 32.4613 | 0.97087 |
| Linoleic acid | 281.2469 | | 400.6370 | 0.97022 |
| 2,4,7-Decatrienoic acid | 167.1065 | | 250.8110 | 0.96554 |
| Salvianolic acid D | 341.0649 | | 217.7890 | 0.96266 |
| Pyrrole-2-carboxylic acid | 112.0392 | | 105.4760 | 0.96237 |
| 10-Hydroxy-2,8-decadiene-4,6-diynoic acid | 177.0546 | | 178.5125 | 0.96216 |
| Fluvoxamine acid | 319.1297 | | 431.9815 | 0.95858 |
| Salvianolic acid G | 419.0964 | | 221.2140 | 0.95503 |
| Nicotinic acid | 124.0393 | | 54.5503 | 0.95490 |
| Benzoic acid | 123.0437 | | 294.3070 | 0.95444 |
| Phenylacetic acid | 137.0596 | | 267.2630 | 0.95333 |
| 4-Quinolinecarboxylic acid | 174.0548 | | 113.7720 | 0.95091 |
| 4-oxo-9Z,11Z,13E,15E-octadecatetraenoic acid | 291.1951 | | 453.6160 | 0.94895 |
| Choline | 31.4941 | | 104.1068 | 0.99964 |
| L-Carnitine | 32.9350 | | 162.1123 | 0.99941 |
| Pyridoxamine | 151.2050 | | 169.0970 | 0.96420 |
| Triacanthine | 179.0520 | | 204.1229 | 0.99941 |
| Shihunine | 170.7810 | | 204.1017 | 0.99961 |
| Pilocarpidine | 168.6485 | | 195.1128 | 0.99759 |
| Cichorine | 70.6163 | | 194.0810 | 0.98171 |
| Tovophyllin B | 132.3830 | | 461.1981 | 0.96518 |
| (alpha)-2-(2-Furanyl)pyrrolidine | 115.0400 | | 138.0913 | 0.96330 |
| Echinopsine | 182.2430 | | 160.0850 | 0.95757 |
| SANGUINARINE | 319.8390 | | 332.0885 | 0.95707 |
| Adifoline | 348.6590 | | 425.1349 | 0.95419 |
| Serratine | 363.4230 | | 280.1901 | 0.94903 |
| Formononetin | 318.0715 | | 269.0803 | 0.99950 |
| Eucalyptin | 344.4815 | | 327.1218 | 0.99875 |
| Porritoxin | 207.1440 | | 306.1695 | 0.98938 |
| Coumarin | 191.1580 | | 147.0440 | 0.98673 |
| Matteuorien | 298.5590 | | 283.0959 | 0.98505 |
| Aesculetin | 156.8220 | | 179.0338 | 0.97416 |
| Gyromitrin | 455.5315 | | 101.0709 | 0.96432 |
| (-)-Amurensisin | 220.5450 | | 441.0783 | 0.94806 |
| Carnosol | 338.6450 | | 331.1877 | 0.99720 |
| alpha-Zearalanol | 251.9665 | | 323.1845 | 0.98009 |
| 2-Nitrophenol | 191.7880 | | 140.0342 | 0.95616 |
| Ineketone | 289.6990 | | 319.2259 | 0.99368 |
| JUGLONE | 275.4180 | | 175.0388 | 0.99010 |
| Risperidone | 205.6595 | | 176.1069 | 0.98734 |
| Normethadone | 381.3265 | | 296.1967 | 0.96956 |
| Drimenin | 347.0430 | | 235.1689 | 0.96106 |
| Tangeritin | 366.1810 | | 373.1271 | 0.95111 |
| Ohobanin | 388.0920 | 283.1324 | | 0.98977 |
